# The mechanism of activation of IRAK1 and IRAK4 by interleukin-1 and Toll-like receptor agonists

**DOI:** 10.1042/BCJ20170097

**Published:** 2017-06-06

**Authors:** Stefan Vollmer, Sam Strickson, Tinghu Zhang, Nathanael Gray, Katherine L. Lee, Vikram R. Rao, Philip Cohen

**Affiliations:** 1 MRC Protein Phosphorylation and Ubiquitylation Unit, Sir James Black Centre, University of Dundee, Dundee DD1 5EH, U.K.; 2 Dana Farber Cancer Institute, Harvard University, Boston, MA, U.S.A.; 3 Worldwide Medicinal Chemistry, Pfizer Research, Cambridge, MA, U.S.A.; 4 Inflammation and Immunology Research Unit, Pfizer Research, Cambridge, MA, U.S.A.

**Keywords:** innate immunity, IRAK, MyD88, Pellino, Toll-like receptor

## Abstract

We have developed the first assays that measure the protein kinase activities of interleukin-1 receptor-associated kinase 1 (IRAK1) and IRAK4 reliably in human cell extracts, by employing Pellino1 as a substrate in conjunction with specific pharmacological inhibitors of IRAK1 and IRAK4. We exploited these assays to show that IRAK4 was constitutively active and that its intrinsic activity towards Pellino1 was not increased significantly by stimulation with interleukin-1 (IL-1) in IL-1R-expressing HEK293 cells, Pam_3_CSK_4_-stimulated human THP1 monocytes or primary human macrophages. Our results, in conjunction with those of other investigators, suggest that the IL-1-stimulated *trans*-autophosphorylation of IRAK4 is initiated by the myeloid differentiation primary response gene 88-induced dimerization of IRAK4 and is not caused by an increase in the intrinsic catalytic activity of IRAK4. In contrast with IRAK4, we found that IRAK1 was inactive in unstimulated cells and converted into an active protein kinase in response to IL-1 or Pam_3_CSK_4_ in human cells. Surprisingly, the IL-1-stimulated activation of IRAK1 was not affected by pharmacological inhibition of IRAK4 and not reversed by dephosphorylation and/or deubiquitylation, suggesting that IRAK1 catalytic activity is not triggered by a covalent modification but by an allosteric mechanism induced by its interaction with IRAK4.

## Introduction

The MyD88 (myeloid differentiation primary response gene 88) signalling network plays a central role in defence against infection by pathogens (reviewed in ref. [1]). The interaction of interleukin-1 (IL-1) with its receptor (IL-1R) or pathogen-associated molecular patterns with Toll-like receptors (TLRs) induces the recruitment of the adaptor protein MyD88 to the receptors, which is followed by the recruitment of interleukin receptor-associated kinase 4 (IRAK4) to MyD88 via interactions between the N-terminal death domains of these proteins. The receptor, MyD88 and IRAK4 form an oligomeric complex, termed the Myddosome, which acts as a platform for the recruitment of the other members of the IRAK family, termed IRAK1, IRAK2 and IRAK3 (IRAK3 is also called IRAKM) [2,3]. IRAK1 and IRAK4 are catalytically active protein kinases, whereas IRAK2 and IRAK3 appear to be inactive pseudokinases [4–6].

Although IRAK1 and IRAK4 were identified many years ago, the mechanisms by which they are activated *in vivo* are still incompletely understood. IRAK1 becomes extensively phosphorylated and ubiquitylated [7] within minutes of the MyD88 signalling network being activated, but whether the catalytic activity of IRAK4 is required for the activation of IRAK1, or even whether the activation of IRAK1 requires its covalent modification has not been established (reviewed in ref. [4]).

Information about the physiological substrates of IRAK1 and IRAK4 is also limited. IRAK4 undergoes *trans*-autophosphorylation when the MyD88-dependent signalling network is activated [8,9], but, to our knowledge, no other physiological substrates for IRAK4 have been validated genetically. The only well-authenticated physiological substrate for IRAK1 is Pellino1. The IRAK1-catalyzed phosphorylation of Pellino1 at multiple amino acid residues *in vitro* converts it from an inactive into an active E3 ubiquitin ligase [10–12]. The IL-1-stimulated activation of Pellino1 in human cells is prevented by pharmacological inhibition of IRAK1 and is reduced in embryonic fibroblasts from knock-in mice expressing the catalytically inactive IRAK1[D359A] mutant [13].

Here, we have used recently developed pharmacological inhibitors of IRAK1 [13,14] and IRAK4 [15] to develop reliable assays for these protein kinases in cell extracts using Pellino1 as a substrate. The further exploitation of these assays has allowed us to make some unexpected findings about the acute regulation of IRAK4 and IRAK1 activities in cells.

## Materials and methods

### Materials

JNK (c-Jun N-terminal Kinase)-IN-7 [14], JNK-IN-8 [14] and IRAK4-IN-1 [15] were synthesized as described, and their structures are shown in Supplementary Figure S1. These compounds were stored at −20°C as 10 mM solutions in dimethyl sulphoxide. The TLR1/2 agonist Pam_3_CSK_4_ was purchased from Invivogen. The IRAK4 inhibitor, 1-{[(2*S*)-5-oxopyrrolidin-2-yl]methoxy}-7-(propan-2-yloxy)isoquinoline-6-carboxamide, herein referred to as IRAK4-IN-1, was synthesized as described recently [15].

### Proteins, antibodies and DNA constructs

Human IL-1β was expressed and purified as described previously [16]. Human Pellino1 and human protein phosphatase-1γ (PP1γ) were expressed in *Escherichia coli* as glutathione-*S*-transferase (GST) fusion proteins and purified by affinity chromatography on glutathione-Sepharose by the Protein Production Team of the MRC Protein Phosphorylation and Ubiquitylation Unit (MRC-PPU), University of Dundee. The catalytic domain of rat USP2 (ubiquitin-specific protease 2) was expressed in *E. coli* and purified by Dr Richard Ewan (MRC-PPU), while phage λ phosphatase was purchased from New England Biolabs.

Antibodies that immunoprecipitate IRAK1 or IRAK4 were raised in sheep and the anti-sera affinity was purified on an antigen-agarose column. The IRAK1 antibody (sheep S690, 3rd bleed) was raised against the full-length mouse protein and the IRAK4 antibody (sheep S522C, 2nd bleed) was raised against the full-length human protein by the Antibody Production Team of the MRC-PPU at Dundee. Immunoblotting was performed with phospho-specific antibodies that recognize p105/NF-κB1 (nuclear factor kappa B) phosphorylated at Ser933, IRAK4 phosphorylated at Thr345/Ser346 and p38α MAP (mitogen-activated protein) kinase phosphorylated at Thr180 and Tyr182. These antibodies, as well as antibodies that recognize all forms of p38α MAP kinase and glyceraldehyde-3-phosphate dehydrogenase (GAPDH), were purchased from Cell Signaling Technology. Antibodies recognizing JNK phosphorylated at Thr183 and Tyr185 or all forms of JNK were from Invitrogen, while anti-IRAK1 for immunoblotting was obtained from Santa Cruz and anti-IRAK4 for immunoblotting from Merck-Millipore. A rabbit secondary antibody conjugated to horseradish peroxidase was from Pierce.

DNA clones encoding HA-IRAK1 (DU8246) and HA-IRAK1[C302L] (DU43693) were inserted into pCMV5 vectors. The proteins, antibodies and DNA clones generated for the present study have been given assigned [DU] numbers and can be ordered from the reagents section of the MRC-PPU website (https://mrcppureagents.dundee.ac.uk/).

### Cell culture and cell stimulation

HEK293 cells stably expressing the IL-1 receptor (IL-1R cells) and IRAK1-null IL-1R cells (kindly provided by Drs Xiaoxia Li and George Stark, Cleveland Clinic, OH, U.S.A.) were cultured in Dulbecco's modified Eagle's medium (DMEM), and the human monocyte cell line THP-1 in RPMI medium, both supplemented with 10% foetal bovine serum, 2 mM l-glutamine and antibiotics (100 Units/ml penicillin and 100 µg/ml streptomycin). Buffy coats were obtained from the East of Scotland Blood Transfusion Centre, Ninewells Hospital, Dundee, U.K. Human peripheral blood mononuclear cells were isolated from the buffy coat by density gradient centrifugation using a Ficoll gradient. The cells were purified by magnetic labelling using CD14 MicroBeads (MACS, Milenyi Biotec). Four million cells were seeded in DMEM supplemented with 10% foetal bovine serum, 2 mM l-glutamine and antibiotics (100 Units/ml penicillin and 100 µg/ml streptomycin) on a 10 cm diameter cell culture dish and differentiated for 7 days into primary human macrophages with recombinant human macrophage colony stimulating factor (0.1 µg/ml) from R&D Systems.

All cells were grown under standard conditions (5% CO_2_, 37°C, water-saturated atmosphere). The cells were incubated for 1 h with or without protein kinase inhibitors prior to stimulation with agonists. IL-1R cells were stimulated for the times indicated in figure legends with 5.0 ng/ml IL-1β and THP-1 cells and human macrophages with 1.0 µg/ml Pam_3_CSK_4_. The medium was removed, and the IL-1R cells or the primary human macrophages washed once with ice-cold PBS followed by lysis in 50 mM Tris–HCl (pH 7.5), 1 mM EGTA, 1 mM EDTA, 1% (v/v) Triton X-100, 1 mM sodium orthovanadate, 50 mM NaF, 5 mM sodium pyrophosphate, 0.27 M sucrose, 10 mM sodium 2-glycerophosphate, 0.2 mM phenylmethylsulphonyl fluoride and 1 mM benzamidine. THP-1 suspension cells were harvested by centrifugation (524×***g*** for 4 min), and the cells were washed and lysed as described for IL-1R cells. The cell lysates were clarified by centrifugation at 13 300×***g*** for 15 min at 4°C, and the supernatants (cell extracts) were removed and either used immediately or stored frozen in aliquots at −80°C until use. Protein concentrations were determined by the Bradford procedure.

### Immunoprecipitation of IRAK1 and IRAK4 and immunoblotting

To immunoprecipitate IRAK1 or IRAK4, 0.5 mg of cell extract protein was incubated for 1 h on a rotating wheel at 4°C with 0.5 µg of antibody. The samples were then incubated for a further hour on a rotating wheel with 15 µl (packed beads) Protein-G Sepharose. Under these conditions, ∼90% of the IRAK1 or IRAK4 was depleted from the cell extracts (Supplementary Figure S1). The beads were collected by brief centrifugation (1 min, 2000×***g****,* 4°C) and washed three times at 4°C with 0.5 ml of lysis buffer containing 0.5 M NaCl, followed by three more washes with 0.5 ml of lysis buffer. Proteins were released from the Protein G-Sepharose beads by incubation for 5 min at 75°C in 1% (w/v) SDS, subjected to SDS–PAGE and transferred to PVDF membranes, and, after blocking with 5% (w/v) non-fat dried milk in 50 mM Tris–HCl (pH 7.5), 0.15 M NaCl and 0.1% (v/v) Tween 20, immunoblotting was performed using the ECL system (GE Healthcare). Immunoblotting of other proteins was carried out in the same way after denaturation of 20 µg of cell extract protein in SDS followed by SDS–PAGE.

### Measurement of IRAK1 and IRAK4 activity in immunoprecipitates

The IRAK proteins were immunoprecipitated as described above, except that the final three washes were carried out in 50 mM Tris–HCl (pH 7.5), 0.1 mM EGTA, 2 mM dithiothreitol and 10 mM magnesium acetate (termed kinase assay buffer). The beads were resuspended in kinase assay buffer containing 0.5 mM [γ-^32^P]ATP (specific radioactivity 2000 cpm/pmol) and GST-Pellino1 (0.5 µg/reaction) in the presence or absence of the protein kinase inhibitors specified in the figure legends. After incubation for 30 min at 30°C with continuous agitation, the reactions were terminated by denaturation in 1% (w/v) SDS, subjected to SDS–PAGE, transferred to PVDF membranes and autoradiographed using Amersham Hyperfilm (GE Healthcare) to detect phosphorylated Pellino1.

### Incubation with phosphatases and deubiquitylases

IRAK proteins were immunoprecipitated from the cell extracts, and the immunoprecipitates were washed three times at 4°C with 0.5 ml of cell lysis buffer containing 0.5 M NaCl and then twice with 0.5 ml of 50 mM Tris–HCl (pH 7.5), 0.05 M NaCl and 5.0 mM dithiothreitol. The immunoprecipitated IRAKs were then incubated for 1 h at 30°C on a rotating platform in 30 µl of 50 mM Tris–HCl (pH 7.5), 0.05 M NaCl, 5.0 mM dithiothreitol containing PP1γ (10 U) or phage λ phosphatase (50 U) and/or the deubiquitylase USP2 (1.15 µg). Where indicated, PP1γ was inactivated by incubation with microcystin (10 µM) prior to addition to the immunoprecipitated IRAKs.

## Results and discussion

### Measurement of endogenous IRAK4 activity in IL-1R cell extracts

We used HEK293 cells that stably express the IL-1 receptor (IL-1R cells) for most of the experiments described in the present paper, because the IL-1-stimulated activation of IκB kinase α (IKKα), IKKβ and MAP kinases is completely dependent on the expression of IRAK1 in these cells [17,18]. IL-1R cells are therefore a simple model system in which to study how IRAK1 and IRAK4 are activated and their roles in the MyD88 signalling network.

**Figure 1.**
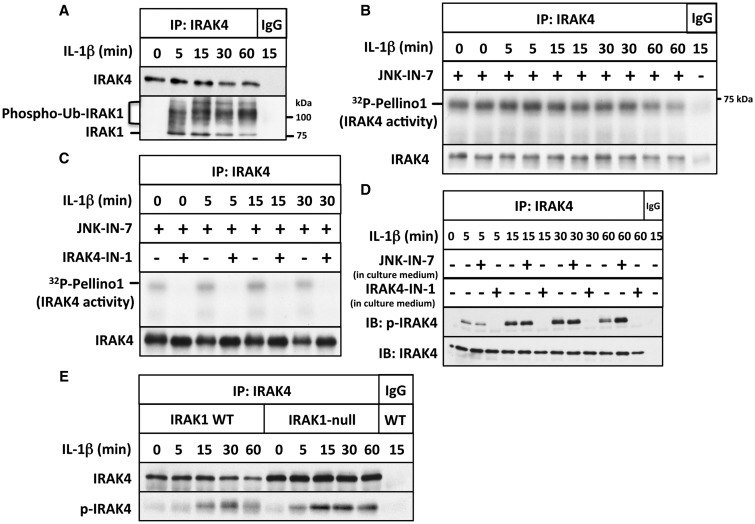
IL-1 stimulates the autophosphorylation but not the activation of IRAK4. (**A**) IL-1R cells were stimulated with IL-1β for the times indicated; IRAK4 immunoprecipitated from 0.5 mg of cell extract protein using anti-IRAK4 or control preimmune IgG. The presence of IRAK4 and that of IRAK1 were detected by immunoblotting, and phosphorylated and ubiquitylated forms of IRAK1 are denoted by phospho-Ub-IRAK1. (**B**) Same as in **A**, except that 1 mg of cell extract protein was used and the immunoprecipitates were assayed for IRAK4 activity using GST-Pellino1 and Mg[γ^32^P-ATP] as substrates in the presence (+) or absence (−) of the IRAK1 inhibitor JNK-IN-7 (1 µM). The figure shows an autoradiograph of the ^32^P-labelled Pellino1 formed during the assay. The radioactive band migrating more rapidly than GST-Pellino1 is a minor proteolytic fragment present in the preparation. The membranes were also immunoblotted for IRAK4. (**C**) Same as in **B**, except that IRAK4 was assayed in the presence of JNK-IN-7 (1 µM) and in the absence (−) or presence (+) of the IRAK4 inhibitor IRAK4-IN-1 (1 µM). (**D**) Same as in **A**, except that the cells were incubated for 1 h in the absence (−) or presence (+) of JNK-IN-7 (10 µM) or IRAK4-IN-1 (3 µM) prior to stimulation with IL-1β, and immunoblotting was carried out with antibodies that recognize all forms of IRAK4 (IRAK4) or antibodies that recognize IRAK4 phosphorylated at Thr345 and Ser346 (p-IRAK4). (**E**) Same as in **D**, except that IRAK4 was immunoprecipitated from both IL-1R cells and IRAK1-null IL-1R cells and no inhibitors were present in the cell culture medium.

The stimulation of IL-1R cells with IL-1 induced the interaction of IRAK4 with IRAK1 and the conversion of IRAK1 into a variety of more slowly migrating species (Figure 1A). These comprise phosphorylated and ubiquitylated species as shown by their reconversion into the unmodified form of IRAK1 by incubation with the protein phosphatase from bacteriophage λ (λPPase) and the USP2. Incubation with both of these enzymes was needed to collapse the more slowly migrating forms of IRAK1 to the unmodified form of IRAK1 that is present in cells not stimulated with IL-1 (Supplementary Figure S2A). Importantly, the IL-1β-dependent interaction of IRAK1 with IRAK4 was sustained for at least 60 min (Figure 1A and Supplementary Figure S2B).

JNK-IN-7 and JNK-IN-8 were originally developed as potent covalent low nanomolar inhibitors of JNK isoforms [14]. These compounds are closely related in structure, but whereas JNK-IN-7 also inhibits IRAK1, JNK-IN-8 does not. Neither compound inhibits IRAK4. We therefore exploited these compounds to investigate aspects of the regulation of IRAK1 and IRAK4. In particular, we included JNK-IN-7 when assaying IRAK4 activity in immunoprecipitates, in order to prevent co-immunoprecipitating IRAK1 from interfering with the measurement of IRAK4 activity. Using Pellino1 as a substrate, we observed that immunoprecipitated IRAK4 was already active under basal conditions and that its activity did not change significantly after stimulation with IL-1 (Figure 1B). The kinase activity towards Pellino1 remaining in the presence of JNK-IN-7 was completely inhibited by the further inclusion of the IRAK4-specific inhibitor IRAK4-IN-1 [15] in the assays (Figure 1C), demonstrating that it was due to IRAK4. Thus, IRAK4 activity could be measured reliably in cell extracts, provided that IRAK1 was inhibited. If JNK-IN-7 was omitted from the assays, much higher levels of Pellino1 kinase activity were measured in the IRAK4 immunoprecipitates, which could be suppressed by JNK-IN-7 (Supplementary Figure S3A). Thus, the inclusion of an IRAK1 inhibitor in the assays was essential for the accurate measurement of IRAK4 activity in these cells.

IRAK4 underwent a relatively slow phosphorylation at both Thr345 and Ser346 in response to IL-1, which was maximal after 15–30 min (Figure 1D). Interestingly, the IL-1-stimulated phosphorylation of IRAK4 at Thr345/Ser346 was suppressed when the IRAK4 inhibitor IRAK4-IN-1 was included in the cell culture medium, but was unaffected by JNK-IN-7 up to 30 min, and even modestly enhanced after 60 min (Figure 1D). These results confirm that the phosphorylation of IRAK4 at Thr345/Ser346 is an autophosphorylation event catalyzed by IRAK4 itself. IRAK4 activity towards Pellino1 was not increased significantly by stimulation with IL-1 (Figure 1B,C), indicating that the unphosphorylated form of IRAK4 is active. However, the possibility that activity increases during the assay as a result of autophosphorylation, as reported for the purified recombinant enzyme [9], cannot be excluded. Other investigators reported that interaction with MyD88 induces the dimerization of IRAK4 [8], which may permit the *trans*-autophosphorylation of IRAK4 to be initiated independently of changes in the intrinsic catalytic activity of IRAK4 [8,9].

The expression of IRAK4 was enhanced in IRAK1 knockout (KO) IL-1R cells, and the IL-1-stimulated autophosphorylation of IRAK4 was still observed in IRAK1 KO IL-1R cells (Figure 1E). Thus, the dimerization of IRAK4 and its ability to undergo *trans*-autophosphorylation is independent of the interaction of IRAK4 with IRAK1. The enhanced expression of IRAK4 in IRAK1 KO cells suggests that IRAK1, or perhaps another protein kinase activated ‘downstream’ from IRAK1, may restrict the expression and/or the stability of IRAK4.

### IRAK4 is constitutively active in other human cells

The finding that IRAK4 was constitutively active in IL-1R cells raised the question of whether this was also true in other human cells. We therefore repeated the experiments shown in Figure 1C using the human THP1 monocyte cell line (Supplementary Figure S4A,B) and primary human macrophages (Supplementary Figure S4C,D). These experiments established that IRAK4 was also active in these cells prior to stimulation of the MyD88-dependent signalling network with Pam3CSK4, an agonist of the TLR1/2 heterodimer (Supplementary Figure S4).

### Measurement of the endogenous IRAK1 kinase activity in IL-1R cell extracts

**Figure 2.**
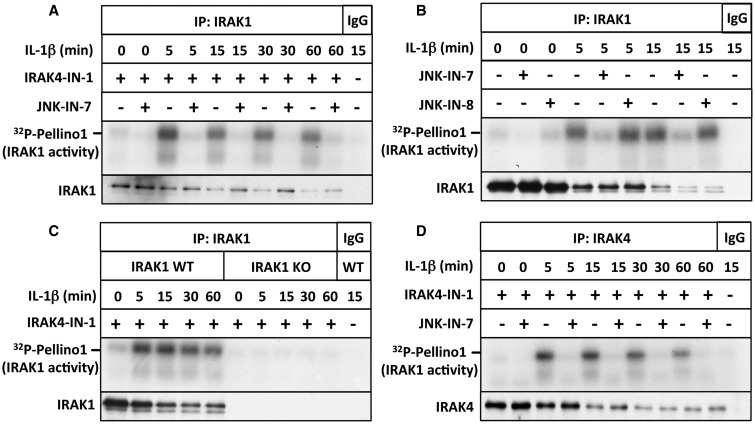
IL-1 induces the activation of IRAK1. (**A**) Same as in Figure 1B, except that IRAK1 was immunoprecipitated with anti-IRAK1 or control IgG and the IPs were assayed for IRAK1 activity using GST-Pellino1 and Mg[γ^32^P-ATP] as substrates in the presence (+) of IRAK4-IN-1 to inhibit co-immunoprecipitating IRAK4, and in the absence (−) or presence (+) of the IRAK1 inhibitor JNK-IN-7 (1 µM). (**B**) Same as in **A**, except that IRAK1 was assayed in the absence (−) or presence (+) of JNK-IN-7 (1 µM) or JNK-IN-8 (1 µM), and the membranes were additionally immunoblotted for all forms of IRAK1 — note that the unmodified form of IRAK1 disappears as it is gradually converted into more slowly migrating phosphorylated and ubiquitylated species after stimulation with IL-1β. (**C**) Same as in **B**, except that IRAK1 was immunoprecipitated from extracts of both IL-1R cells and IRAK1 KO IL-1R cells, and JNK-IN-7 was omitted from all assays. (**D**) Same as in **B**, except that IRAK4 was immunoprecipitated, and IRAK1 in the immunoprecipitates was assayed by including IRAK4-IN-1 in the assays.

To measure IRAK1 catalytic activity, we immunoprecipitated this protein kinase from the cell extracts and assayed it using Pellino1 as a substrate. As IRAK4 was co-immunprecipitated with IRAK1 in IL-1-stimulated cells (but not in cells not stimulated with IL-1) (Supplementary Figure S5), we included the IRAK4 inhibitor IRAK4-IN-1 in IRAK1 assays to avoid interference from IRAK4 activity. Under these conditions, all the Pellino1 kinase activity associated with IRAK1 immunoprecipitates was suppressed by the IRAK1 inhibitor JNK-IN-7 (Figure 2A), but not by the control compound JNK-IN-8 (Figure 2B), which does not inhibit IRAK1. Thus, the Pellino1 kinase activity, measured in the presence of IRAK4-IN-1, assays IRAK1 catalytic activity specifically and is not influenced by JNK isoforms that might be present as trace contaminants, which are potently inhibited by both JNK-IN-7 and JNK-IN-8 [14]. The specificity of the assay was confirmed by experiments with IRAK1 KO IL-1R cells. No IRAK1 activity could be detected in the extracts of these cells after immunoprecipitation with anti-IRAK1 (Figure 2C).

IRAK1 had little activity in cells not stimulated with IL-1, but was converted into an active form within 5 min of IL-1 stimulation. The activity peaked after 5–15 min in IL-1R cells and declined modestly thereafter (Figure 2A). If the IRAK4 inhibitor IRAK4-IN-1 was omitted, trace Pellino1 kinase activity could be detected in the presence of JNK-IN-7 (Supplementary Figure S3B), indicating that IRAK4 makes a minor contribution to the total Pellino1 kinase activity associated with IRAK1 immunoprecipitates.

It is noteworthy that IRAK1 can also be assayed in IRAK4 immunoprecipitates if the IRAK4 inhibitor IRAK4-IN-1 is included in the assays (Figure 2D). Thus, IRAK1 and IRAK4 catalytic activities can be measured simultaneously in IRAK4 immunoprecipitates by including appropriate pharmacological inhibitors in the assays.

### The IL-1-stimulated activation of IRAK1 does not require IRAK4 catalytic activity

**Figure 3.**
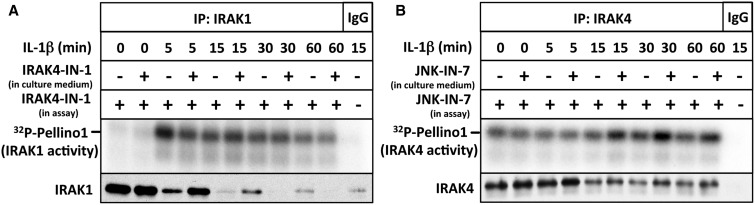
The IL-1-stimulated activation of IRAK1 does not require the catalytic activity of IRAK4. (**A**) IL-1R cells were incubated for 1 h without (−) or with (+) 3 µM IRAK4-IN-1 to inhibit cellular IRAK4 and stimulated with IL-1β for the times indicated. IRAK1 was immunoprecipitated from 1 mg of cell extract protein with anti-IRAK1 or control IgG and assayed with GST-Pellino1 and Mg[γ^32^P-ATP] as substrates in the presence (+) of 1 µM IRAK4-IN-1. Further details are given in Figure 2A. (**B**) Same as in **A**, except that the cell culture medium was incubated with 10 µM JNK-IN-7 to inactivate cellular IRAK1 activity, and IRAK4 was immunoprecipitated from the cell extracts instead of IRAK1, and co-immunoprecipitating IRAK4 was assayed by including JNK-IN-7 in the assay to inhibit IRAK1.

To investigate whether IRAK4 kinase activity was needed for the IL-1-stimulated activation of IRAK1, we incubated IL-1R cells in the presence or absence of the IRAK4 inhibitor IRAK4-IN-1 prior to stimulation with IL-1. Following IL-1-stimulation, IRAK1 was immunoprecipitated from the cell extracts and assayed in the presence of IRAK4-IN-1. The concentration of IRAK4-IN-1 added to the cells (3 µM) was sufficient to prevent the IL-1-stimulated autophosphorylation of IRAK4 (Figure 1D). These experiments revealed that the presence of the IRAK4 inhibitor in the culture medium had little effect on the IL-1-dependent activation of IRAK1 (Figure 3A). Thus, IRAK4 catalytic activity is not required for the activation of IRAK1.

### The cellular activity of IRAK4 is decreased modestly by IRAK1

To investigate whether IRAK1 activity can influence IRAK4 activity in cells, we incubated IL-1R cells with JNK-IN-7 to irreversibly inactivate IRAK1 (see below) and then immunoprecipitated IRAK4 at different times after IL-1 stimulation. These experiments showed that, similar to the autophosphorylation of IRAK4 at Thr345 and Ser346 (Figure 1D), the Pellino1 kinase activity of IRAK4 was also increased slightly after prolonged IL-1-stimulation whenever IRAK1 was inactivated by the addition of JNK-IN-7 to the culture medium (Figure 3B). This suggests that IRAK1 and/or JNK activity may modestly reduce the activity of IRAK4. This is consistent with the enhanced IL-1-dependent autophosphorylation of IRAK4 in IRAK1-null IL-1R cells (Figure 1E).

### JNK-IN-7 irreversibly inactivates IRAK1 by covalent modification of Cys302

**Figure 4.**
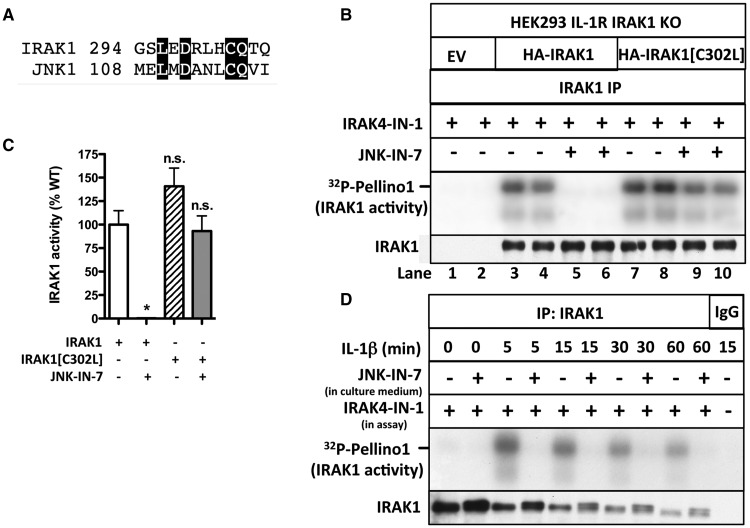
JNK-IN-7 is an irreversible inhibitor of IRAK1. (**A**) The amino acid sequences of human IRAK1 (residues 294–305) and human JNK1 (residues 108–119) were aligned, and conserved residues are highlighted. Met108 of JNK1 is the ‘gatekeeper’ residue. (**B**) IRAK1 KO IL-1R cells were transfected with 5 µg or DNA of a control empty vector (EV) (lanes 1 and 2), HA-tagged wild-type IRAK1 (lanes 3–6) or HA-tagged IRAK1[C302L] (lanes 7–10). IRAK1 was immunoprecipitated from 0.5 mg of cell extract protein and assayed with GST-Pellino1 and Mg[γ^32^P-ATP] in the presence of IRAK4-IN-1 to inhibit co-immunoprecipitating IRAK4 and in the presence or absence of the IRAK1 inhibitor JNK-IN-7. (**C**) The autoradiogram from **B** and two other independent experiments were scanned, and the activity of IRAK1 and IRAK1[C302L] (IRAK1[C/L]) measured without or with 1.0 µM JNK-IN-7. The results are shown as a % of wild-type IRAK1 activity in the absence of JNK-IN-7. **(D**) IL-1R cells were incubated for 1 h without (−) or with (+) 10 µM JNK-IN-7 to inhibit cellular IRAK1 and then stimulated with IL-1β. IRAK1 was immunoprecipitated from 1 mg of cell extract protein and assayed with GST-Pellino1 and Mg[γ^32^P-ATP] as substrates in the presence (+) of 1 µM IRAK4-IN-1 to inhibit co-immunoprecipitating IRAK4.

JNK-IN-8 inactivates c-Jun N-terminal kinases 1 and 2 (JNK1 and JNK2) by covalent modification of Cys153 in the catalytic domain [14]. Cysteine302 in human IRAK1 is the amino acid residue equivalent to Cys153 of JNK1 (Figure 4A). To investigate whether JNK-IN-7 modifies IRAK1 covalently, we mutated Cys302 to Leu. DNA vectors encoding HA-tagged wild-type IRAK1 and the IRAK1[C302L] mutant were transfected into IRAK1-null IL-1R cells and assayed after their immunoprecipitation from the cell extracts. These experiments showed that wild-type IRAK1 was inactivated by JNK-IN-7, but the IRAK1[C302L] mutant was much less affected (Figure 4B,C). These results suggest that the potency with which JNK-IN-7 inhibits IRAK1 is enhanced by the covalent modification of Cys302. Interestingly, Cys302 of human IRAK1 is replaced by Leu in murine IRAK1, suggesting that JNK-IN-7 may be a weak inhibitor of mouse IRAK1 and that the use of JNK-IN-7 as an IRAK1 inhibitor may be confined to human cells.

To confirm that JNK-IN-7 inactivates human IRAK1 irreversibly, we incubated IL-1R cells with JNK-IN-7 and immunoprecipitated IRAK1 from the lysates of IL-1-stimulated cells. After washing extensively to remove free, unreacted JNK-IN-7, we assayed IRAK1 activity using Pellino1 as a substrate. These experiments revealed that incubation of the cells with JNK-IN-7 had either inactivated IRAK1 irreversibly or had prevented its activation by IL-1 (Figure 4D). The experiments presented in the following section indicate that the former interpretation is correct.

### Evidence that IRAK1 activity is not affected significantly by phosphorylation or ubiquitylation

**Figure 5.**
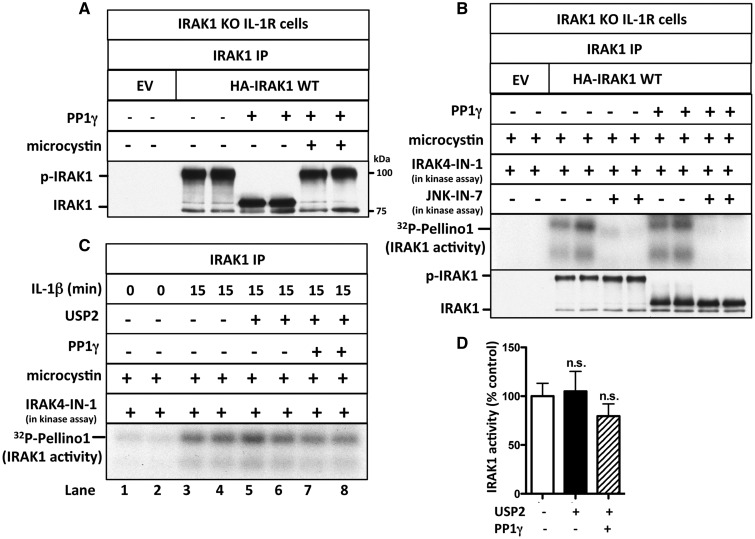
IRAK1 activation does not require its phosphorylation or ubiquitylation. (**A**) IRAK1 KO IL-1R cells were transfected with 5 µg of DNA of control empty vector (EV) or HA-IRAK1, and IRAK1 then immunoprecipitated from 0.5 mg of cell extract protein. The IPs were incubated with PP1γ (10 U) in the presence or absence of microcystin (10 µM) and denatured in SDS. Following SDS–PAGE and transfer to PVDF membranes, immunoblotting was performed with anti-IRAK1. (**B**) As in **A**, except that, after PP1γ treatment, the immunoprecipitates were washed, incubated for 1 h with 10 µM microcystin, and IRAK1 was assayed with GST-Pellino1 and Mg[γ^32^P-ATP] as substrates in the absence (−) or presence (+) of JNK-IN-7 (1 µM) and in the presence (+) of IRAK4-IN-1 (1 µM) to inactivate co-immunoprecipitating IRAK4. The presence of IRAK1 in the immunoprecipitates was also analyzed by immunoblotting. (**C**) IL-1R cells were stimulated with IL-1β and IRAK1 immunoprecipitated from 1 mg of cell extract protein and incubated with PP1γ (10 U) and USP2 (1.15 µg). The immunoprecipitates were washed, incubated for 1 h with 1 µM microcystin to inactivate any residual PP1γ, and IRAK1 assayed with GST-Pellino1 and Mg[γ^32^P-ATP] as substrates in the presence (+) of IRAK4-IN-1 (1 µM) to inactivate any co-immunoprecipitating IRAK4. (**D**) The autoradiogram from **C** and one other independent experiment were scanned and IRAK1 activity quantitated after incubation with or without USP2 and PP1γ. The results are presented as a % of that measured without USP2 and PP1γ treatment.

Wild-type human IRAK1 was overexpressed in IRAK1 KO IL-1R cells. The transfected IRAK1 was active even in cells not stimulated with IL-1 and underwent extensive autophosphorylation that resulted in considerable retardation of its mobility on SDS–polyacrylamide gels. Treatment with PP1γ reconverted IRAK1 into the unmodified protein, and this was prevented by the inclusion of the cyclic peptide microcystin in the assays, which is an inhibitor of PP1 and other members of the PPP family of serine/threonine-specific phosphatases (Figure 5A). IRAK1 does not undergo ubiquitylation in these transfection experiments because the cells are not stimulated with IL-1β. The activity of transfected IRAK1 was suppressed by JNK-IN-7, but was not decreased by treatment with PP1γ (Figure 5B). Moreover, only trace reconversion of IRAK1 to the autophosphorylated species occurred during the assays (Figure 5B, bottom panel). These experiments suggest that autophosphorylation of the transfected IRAK1 is a consequence and not a cause of IRAK1 activation.

We next studied the activation of the endogenous IRAK1 by immunoprecipitating it from IL-1β-stimulated IL-1R cells. IL-1-stimulation induced the ubiquitylation, as well as the phosphorylation of IRAK1, and so, we assayed IRAK1 before and after deubiquitylation or deubiquitylation plus dephosphorylation (Figure 5C and see also Supplementary Figure S2A). These experiments indicated that the activity of the unmodified IRAK1 was similar to that of the phosphorylated and ubiquitylated species (Figure 5C,D).

Taken together, the results indicate that the IL-1-dependent interaction of IRAK1 with IRAK4 is sufficient to trigger IRAK1 activation without any covalent modification by phosphorylation or ubiquitylation.

### The role of IRAK4 catalytic activity in the activation of IKKβ and MAP kinases

**Figure 6.**
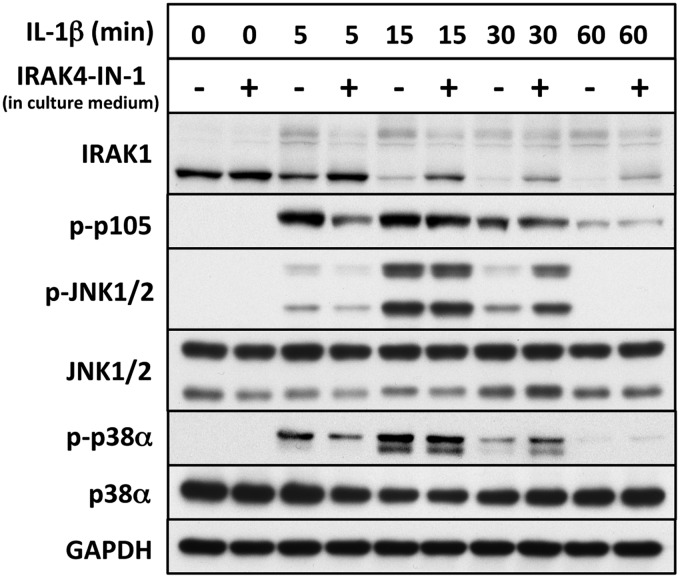
The inhibition of IRAK4 does not prevent the IL-1-stimulated activation of IKKβ and MAP kinases in IL-1R cells. IL-1R cells were incubated for 1 h without (−) or with (+) 3 µM IRAK4-IN-1 to inactivate cellular IRAK4, and then stimulated with IL-1β. Cell extract (20 µg of protein) was subjected to SDS–PAGE and immunoblotting with antibodies recognizing all forms of IRAK1, JNK, p38α MAP kinase and GAPDH, and antibodies that recognize the phosphorylated (p) forms of p105/NF-κB1, JNK1/2 and p38α MAP kinase.

The protein p105/NF-κB1 is a well-authenticated physiological substrate of IKKβ [19,20]. Incubation of IL-1R cells with the IRAK4 inhibitor IRAK4-IN-1 at a concentration that suppresses the IL-1-stimulated autophosphorylation of IRAK4 (Figure 1D) consistently delayed the phosphorylation of p105/NF-κB1 and JNK1/JNK2 by a few minutes, but did not affect the maximal level of phosphorylation attained after 15 or 30 min (Figure 6). Thus, IRAK4 catalytic activity modestly enhances the rate at which IKKβ phosphorylates p105/NF-κB1 and the rate at which JNK1 and JNK2 are activated in these cells over the first 30 min of IL-1 signalling. The inhibition of IRAK4 did not suppress the IL-1-stimulated phosphorylation of p38α MAP kinase (p38) and actually prolonged the activation of both p38α MAP kinase and JNK1/JNK2 (see the 30 min time point in Figure 6). These experiments indicate that IRAK4 catalytic activity is not required to initiate IL-1-signalling in IL-1R cells.

Other investigators reconstituted IRAK4-deficient human fibroblasts with IRAK4 mutants that do not dimerize and autophosphorylate. They observed that the acute IL-1-dependent activation of JNK1/2 and the IKK complex were partially reduced, although phosphorylation of the IKK substrate IκBα was unimpaired (see Figure 5 of [8]). These studies also indicate that neither IRAK4 dimerization nor its autophosphorylation are essential for the signalling events that occur during the first 30 min after stimulation with IL-1, although they may enhance IL-1 signalling.

### Concluding remarks

In the present study, we introduced robust assays to monitor the endogenous IRAK4 and IRAK1 activities in cell extracts, which have permitted these protein kinase activities to be studied independently of one another for the first time. Establishing these assays has provided new insights into the mechanisms by which IRAKs 1 and 4 are activated. We found that IRAK4 is constitutively active and its activity was not increased when the MyD88 signalling network is switched on. Our results show that the agonist-dependent *trans*-autophosphorylation of IRAK4 at Thr345 and Ser346 is not a measure of IRAK4 activity, but is a consequence of the dimerization of IRAK4 triggered by its interaction with MyD88. Moreover, although IL-1-stimulaion converts IRAK1 from an inactive into an active form, the activation of IRAK1 does not require IRAK4 activity, or the phosphorylation or ubiquitylation of IRAK1. It would therefore appear that IRAK1 is activated when it interacts with IRAK4, perhaps by IRAK4-induced IRAK1 dimerization [2,3]. This is consistent with the finding that the IL-1-dependent interaction between IRAK1 and IRAK4 is sustained for at least an hour in IL-1R cells (Figure 1A and Supplementary Figure S2B). IRAK1 is activated without IL-1 stimulation when it is transfected into HEK293 cells (Figure 5B), perhaps because it dimerizes in the absence of IRAK4 at these supraphysiological concentrations. Our results disagree with the report that IRAK1 dissociates from IRAK4 within minutes of stimulation with IL-1 [21]. They also do not support the conclusion that the phosphorylation of IRAK1 by IRAK4 or autophosphorylation enhances the intrinsic catalytic activity of IRAK1 [22]. However, the phosphorylation of one or more threonine residues in IRAK1 enables IRAK1 to interact with the Forkhead-associated domains of Pellino isoforms [23], which is likely to facilitate the IRAK1-catalyzed phosphorylation of these proteins and their conversion into active E3 ligases.

It has been reported that IRAK1 is degraded by the proteasome within minutes of the MyD88 signalling network being activated [24]. However, we found here (Supplementary Figure S1A), and in another study [5], that the ‘disappearance’ of IRAK1 is not caused by its degradation, but by its conversion into a great variety of more slowly migrating phosphorylated and ubiquitylated species that are poorly recognized by some IRAK1 antibodies. Thus, the unmodified form of IRAK1 can be fully recovered by treatment with a phosphatase and a deubiquitylase.

## Abbreviations

DMEM: Dulbecco's Modified Eagle's Medium
GAPDH: glyceraldehyde 3-phosphate dehydrogenase
GST: glutathione-*S*-transferase
IKK: IκB kinase
IL-1: interleukin-1
IL-1R: IL-1 receptor
IRAK: IL-1R-associated kinase
JNK: c-Jun N-terminal Kinase
KO: knockout
MAP: mitogen-activated protein
MRC-PPU: Medical Research Council Protein Phosphorylation and Ubiquitylation Unit
MyD88: myeloid differentiation primary response gene 88
NF-κB: nuclear factor kappa B
PP1: protein phosphatase 1
TLR: Toll-like receptor
USP2: ubiquitin-specific protease 2
